# Negroes and Yellow Fever

**Published:** 1877-10-20

**Authors:** 


NEGROES AND YELLOW FEVER. — In the epidemic of yellow fever, at Savannah, it was said that the fever attacked the blacks with almost equal fatality as the whites. It was discovered, however, that the mulattoes, or mixed breeds, alone suffered, and that the pure negro was exempt from the disease. So it is reported of the fever at Fernandina, Fla., at which point the disease still prevails. The mortality is comparatively light.

